# Influence of Age on the Effectiveness of Lee Silverman Voice Treatment^®^ BIG in Patients with Parkinson’s Disease: A Retrospective Exploratory Observational Study

**DOI:** 10.3390/geriatrics11030063

**Published:** 2026-05-20

**Authors:** Masanobu Iwai, Kazuya Takeda, Soichiro Koyama, Ikuo Motoya, Yuichi Hirakawa, Hiroaki Sakurai, Yoshikiyo Kanada, Nobutoshi Kawamura, Mami Kawamura, Shigeo Tanabe

**Affiliations:** 1 Department of Rehabilitation, Kawamura Hospital, 1-84 Daihannya Akutami, Gifu 501-3144, Japan; 2 Faculty of Rehabilitation, School of Health Sciences, Fujita Health University, 1-98 Dengakugakubo, Toyoake 470-1192, Japan; 3 Department of Neurology, Kawamura Hospital, 1-84 Daihannya Akutami, Gifu 501-3144, Japan

**Keywords:** ADL, gait speed, LSVT^®^ BIG, motor symptoms, QoL, task-based training

## Abstract

**Background/Objectives:** Advanced age in Parkinson’s disease (PD) is linked to worse motor function, more severe symptoms, and impaired activities of daily living (ADLs). Lee Silverman Voice Treatment (LSVT)^®^ BIG may be suitable for older patients, as it can be adapted to individual abilities. This study evaluated whether age affects the effectiveness of LSVT^®^ BIG on gait speed, motor symptoms, ADLs, and quality of life (QoL) in PD. **Methods:** In this retrospective, single-center cohort study, 22 patients with PD were divided into an older group (≥65 years; *n* = 16) and a younger group (<65 years; *n* = 6). All participants completed 16 one-hour, face-to-face LSVT^®^ BIG sessions. Gait speed was assessed with the 10-m walk test; motor symptoms with Movement Disorders Society-Unified Parkinson’s Disease Rating Scale (MDS-UPDRS) Part III; ADLs with MDS-UPDRS Part II; and QoL with the Parkinson’s Disease Questionnaire-39 Summary Index. Two-way mixed-design analysis of variance with aligned rank transformation was used for statistical analysis. **Results:** Significant improvements were observed for all outcomes (gait speed, motor symptoms, ADLs, and QoL) after the intervention. A significant effect of age group was found for gait speed, with younger patients walking faster overall. No significant interaction between timepoint and group was observed for any measure. **Conclusions:** LSVT^®^ BIG appears to improve gait speed, motor symptoms, ADLs, and QoL in patients with PD, regardless of age, suggesting it is an effective intervention for both older and younger patients.

## 1. Introduction

Parkinson’s disease (PD) is a slowly progressive neurodegenerative disorder. Globally, the number of patients with PD is projected to reach 25.2 million by 2050, representing a 112% increase from 2021 [1]. The growth in cases from 2021 to 2050 is predicted to be driven mainly by population aging (89%), followed by population growth (20%) and changes in prevalence (3%) [1]. Regarding the prevalence of PD, it is uncommon before the age of 50; however, its prevalence increases in individuals aged 65 years and older, peaking in the 80–84 age group [1,2]. Geographic disparities exist in the global distribution of PD, with a higher prevalence in developed countries compared to developing countries. Latin America and the Caribbean have the highest prevalence, followed by East Asia and the Pacific, Europe and Central Asia, the Middle East and North Africa, South Asia, and sub-Saharan Africa [3]. Sex differences also exist, with men being more affected, although the difference is relatively modest [3,4].

PD is characterized by motor symptoms, including rigidity, bradykinesia, tremor, postural instability, and gait disorders (postural instability and gait disorder [PIGD]), as well as non-motor symptoms, such as sensory, autonomic, and cognitive-behavioral symptoms [5,6]. Motor impairments are associated with declines in activities of daily living (ADLs) and quality of life (QoL) [7,8]. Rigidity is present in up to 89% of patients with PD [9] and is characterized by increased, velocity-independent muscle tone during passive limb movement [8]. Rigidity interferes with ADLs such as bed mobility and gait [10,11,12,13,14] and is associated with reduced QoL [15]. Bradykinesia is a characteristic clinical feature and is composed of three components: sequence effect, which includes gradual amplitude decrement or slowing during repetitive or continuous voluntary movements; hypokinesia, which includes under-scaled or low-amplitude movements; and akinesia, which includes loss of spontaneous or automatic movements, difficulty initiating movement, or absence of movement [16]. These manifestations result in slow movement and reaction times, leading to impaired performance of ADLs requiring fine motor control, such as buttoning or using utensils. Tremor, a common symptom of PD, is present in approximately 70% of patients [17] and consists of three types: resting tremor, postural tremor, and kinetic tremor [17]. Tremor interferes with ADLs such as handwriting and the use of everyday technology (e.g., smartphones and computers) [18]. In addition, QoL declines due to discomfort and fatigue in the affected body parts, as well as concerns about others’ perceptions when attempting to conceal the tremor [18]. PIGD marks the onset of moderate to severe disease [19]. It is characterized by impaired balance due to loss of postural reflexes, postural deformities such as antecollis, camptocormia, Pisa syndrome, and scoliosis, as well as festination or shuffling gait. PIGD is significantly associated with ADL impairment and declines in QoL [20]. From the perspective of aging and the older population, advanced age among patients with PD is associated with greater severity of motor symptoms and impaired gait and balance ability [21,22,23]. Di Lazzaro et al. (2025) reported that patients with PD onset at ≥65 years exhibit more severe motor symptoms than those with onset before age 65 [22]. De Carolis et al. (2023) reported that patients with PD onset at ≥70 years have more impaired gait and balance ability than those with onset at ≤49 years [23]. In addition, aging itself is associated with declines in physical fitness and performance, including muscle volume, muscle strength, flexibility, speed, and endurance [24,25]. In particular, previous studies have reported that lower-limb physical performance, muscle strength, and muscle quality decrease significantly with age [24]. Age-related declines in physical fitness and performance are associated with ADL impairment and poorer physical-related QoL [26,27,28].

Aging also affects the effectiveness of PD management. Exercise therapies, which are non-pharmacological interventions, have been reported to show varying effectiveness with age [29,30]. A systematic review and network meta-analysis reported that physical therapy, yoga, body-weight-supported treadmill training, resistance training, and multicomponent exercise programs were beneficial for improving motor symptoms in patients aged ≥50 years with PD [29]. However, a meta-analysis of randomized controlled trials reported that structured exercise interventions resulted in significant improvements in gait speed, but not in motor symptoms or QoL, in older patients aged ≥60 years with PD [30].

Lee Silverman Voice Treatment^®^ BIG (LSVT^®^ BIG) is an exercise-based intervention tailored to patients with PD. Conceptually, LSVT^®^ BIG is designed to improve motor symptom such as bradykinesia, hypokinesia, and akinesia [31,32,33] and is grounded in three principles of motor learning: large-amplitude movements, high effort and intensive treatment, and proprioceptive recalibration. The standardized program consists of one-hour sessions, conducted 4 consecutive days a week for 4 weeks, under the guidance of a certified LSVT^®^ BIG therapist. The exercises use external cues, including therapist modeling and tactile, verbal, or visual cues [31,34]. Intervention difficulty is adjusted individually based on patient ability, with adaptations such as handrail support or seated exercises for patients with balance or gait impairments. In addition, the protocol incorporates task-based exercises tailored to individual ADL needs, facilitating the transfer of large-amplitude movements into daily life beyond the treatment setting [31,33,34]. Previous studies have shown that LSVT^®^ BIG improves gait speed, motor symptoms, ADLs, and QoL in patients with PD [35,36].

LSVT^®^ BIG is suitable for older patients with PD who have impaired motor function and severe motor symptoms, as it is tailored to individual abilities. Furthermore, the LSVT^®^ BIG protocol incorporates task-based ADL exercises that accommodate age-related differences in functional and ADL abilities influenced by aging. Therefore, LSVT^®^ BIG may be equally effective in older and younger patients with PD. However, to the best of our knowledge, no studies have examined age-related differences in the effectiveness of LSVT^®^ BIG. The aim of this study was to investigate the impact of age on the effectiveness of LSVT^®^ BIG on gait speed, motor symptoms, ADLs, and QoL in individuals with PD.

## 2. Materials and Methods

### 2.1. Study Design

We conducted a single-center, retrospective cohort study in accordance with the Strengthening Reporting of Observational Studies in Epidemiology guidelines [37].

### 2.2. Participants

Participants were recruited using a convenience sampling method. Data were gathered from inpatients and outpatients through the Kawamura Hospital database between January 2019 and November 2025. The inclusion criteria were: (1) a diagnosis of PD by neurologists; (2) age between 40 and 100 years; (3) PD classified as Hoehn and Yahr (H&Y) stages I to IV; and (4) prior experience with LSVT^®^ BIG as a prescribed therapy based on clinical decisions, rather than for research purposes. The exclusion criteria included: (1) muscular, orthopedic, or neurological conditions (other than PD) that could affect the implementation of LSVT^®^ BIG; (2) moderate to severe non-motor symptoms as indicated by a total score greater than 10 on the Movement Disorders Society-Unified Parkinson’s Disease Rating Scale (MDS-UPDRS) Part I [38]; and (3) missing data. The MDS-UPDRS Part I was included in the exclusion criteria because non-motor symptoms, such as anxiety, fatigue, apathy, difficulty concentrating, and constipation, are associated with reduced physical activity [39,40] and may present barriers to regular exercise in patients with PD.

### 2.3. Patients’ Demographic Characteristics

All assessments were conducted by one or two of the ten LSVT^®^ BIG-certified physical and occupational therapists affiliated with Kawamura Hospital. Demographic characteristics collected included age, sex, time since PD diagnosis, H&Y stage, levodopa equivalent daily dose (LEDD), total score of the MDS-UPDRS Part I, and the Japanese version of the Mini-Mental State Examination (MMSE) score.

The H&Y stage evaluates the severity of overall dysfunction in patients with PD, categorizing severity from stage I to V, where higher stages indicate greater motor dysfunction and ADL impairments [41].

The LEDD represents the total daily dose of antiparkinsonian drugs. Levodopa, the most effective and widely used treatment for PD, poses a risk of developing motor and non-motor complications, such as dyskinesia and motor/non-motor fluctuations, at higher dosages. The LEDD was calculated to quantify dopaminergic medication usage following the method described by Tomlinson et al. (2010) [42].

The MDS-UPDRS provides a comprehensive assessment of clinical symptoms of the disease, including non-motor symptoms (Part I), ADLs (Part II), motor symptoms (Part III), and motor complications (Part IV). All items are scored on a scale from 0 (normal) to 4 (severe), with higher scores indicating more severe disease symptoms. The MDS-UPDRS Part I specifically assesses the non-motor impact of PD on daily living, including mood and sleep disorders, dysautonomia, apathy, fatigue, and pain. It consists of 13 items (six rater-based and seven patient self-assessment), with a maximum score of 52. The MDS-UPDRS Part I demonstrates high internal consistency (Cronbach’s alpha [α] = 0.79) and strong concurrent validity (Pearson’s correlation coefficient [r] = 0.76) in individuals with PD [43].

The MMSE is widely used to screen for cognitive impairment, measuring attention and orientation, memory, registration, recall, calculation, language, and the ability to draw a complex polygon. It consists of 15 items, with a maximum score of 30. All items are scored on a scale from 0 to 2, with lower scores indicating greater cognitive impairment. The MMSE is reliable and demonstrates concurrent validity, with Pearson’s correlation coefficients (r) ranging from 0.83 to 0.99, and r ≥ 0.67 [44].

### 2.4. Intervention

All participants received 16 sessions of face-to-face exercise with certified physical or occupational therapists. Each intervention was conducted by one of the two or three therapists assigned to the individual prior to treatment. Participants were on stable levodopa medication. One-hour sessions occurred four times a week for 4 weeks. The exercise sessions consisted of four parts in the following order of increasing difficulty: standardized whole-body movements, functional tasks, hierarchical tasks, and BIG walking. In the first half, standardized whole-body movements and functional tasks were conducted for 30 min or more. In the latter half, hierarchical tasks and BIG walking were conducted for 30 min or less [32,33].

The standardized whole-body movements included seven daily multidirectional exercises—stepping, reaching, and stretching—performed with maximal amplitude and effort. These exercises involved sustained and 8–20 repetitive whole-body movement patterns. The seven tasks were performed while standing, sitting, or lying down, depending on individual ability. Functional tasks consist of a sit-to-stand movement and four additional functional component tasks tailored to individual needs, focusing on goal-directed ADLs based on the participant’s self-identified movement challenges. The hierarchical tasks consisted of complex, multistep ADL activities, with one to three selected sub-tasks designed to align with the participant’s goals and interests. Throughout the 16 sessions, these sub-tasks were gradually linked to practice the entire activity. BIG walking involves high-amplitude extremity movements and high-effort ambulation over a designated distance and time [32,33].

During the LSVT^®^ BIG intervention, patients received feedback from therapists through verbal, visual, and tactile cues to encourage larger amplitude movements. They were encouraged to strive for a maximum perceived effort level of 7–8 out of 10 using a modified Borg scale. Patients were also encouraged to incorporate larger and more repetitive movements into their daily activities to ensure continuous exercise throughout the day [32,33].

In addition, a home exercise program was implemented on both exercise and non-exercise days, consisting of a condensed version of functional component movements, including BIG walking (i.e., a shorter version of the treatment sessions). On exercise days, participants performed an extra 5–10 min session, while on non-exercise days, they completed two sessions of 10–15 min each. A homework book was provided, and participants were held accountable for completing the exercises [32,33].

### 2.5. Clinical Assessments of Motor Symptoms, ADLs, and QoL

Each patient’s assessments were performed within 1 week before and after the LSVT^®^ BIG intervention by one or two certified physical or occupational therapists affiliated with the hospital who were not involved in the intervention. All assessments were conducted during the “on” medication state to eliminate variability due to medication fluctuations (e.g., on/off state). Assessments included the 10-m walking test (10MWT) for gait speed, MDS-UPDRS Part III for motor symptoms, MDS-UPDRS Part II for ADLs, and the Parkinson’s Disease Questionnaire-39 Summary Index (PDQ-39 SI) for QoL assessment.

The 10MWT was administered in a 10-m corridor, with an additional 2 m at both ends, marked with tape for acceleration and deceleration (14 m total) [45]. Walking aids were allowed during the test. Participants were instructed to walk at a comfortable speed after receiving a verbal command to start. The total time taken to ambulate 10 m was recorded when the lead foot crossed the respective markers. Gait speed was calculated in meters per second (m/s) [45]. The 10MWT has high intra-rater reliability (intraclass correlation coefficient = 0.81) [46] and concurrent and criterion-related validity in individuals with neurological diseases such as PD [47].

MDS-UPDRS Part III assesses motor signs of PD, including bradykinesia, rigidity, tremor, and PIGD. It consists of 33 items, with a maximum score of 132. A therapist provides instructions, demonstrates tasks, and evaluates the patient’s performance. Higher scores indicate more severe motor impairment. The MDS-UPDRS Part III has high internal consistency (Cronbach’s alpha [α] = 0.93) and strong concurrent validity (Pearson’s correlation coefficient [r] = 0.96) in individuals with PD [43].

MDS-UPDRS Part II assesses disability and independence in ADLs (feeding, dressing, hygiene, walking, turning in bed, getting out of a chair, and so on). It consists of 13 items, with a maximum score of 52. The patient’s usual ADL ability over the past week was evaluated. The patient and/or caregiver provided answers, which were supervised and reviewed by a therapist. Higher scores indicate greater ADL impairment. The MDS-UPDRS Part II has high internal consistency (Cronbach’s alpha [α] = 0.90) and strong concurrent validity (Pearson’s correlation coefficient [r] = 0.92) in individuals with PD [43].

The PDQ-39 SI assesses health status and QoL, including 39 questions addressing eight domains: mobility, ADLs, emotional well-being, stigma, communication, social support, cognition, and bodily discomfort. All items are scored from 0 (never) to 4 (always). The PDQ-39 SI is calculated by summing the scores for the eight dimensions and dividing by the total number of dimensions. Scores on the PDQ-39 SI range from 0 (indicating no problems) to 100 (indicating extreme problems), with higher scores indicating lower health-related QoL. The PDQ-39 SI has a high level of internal consistency (α = 0.84) and concurrent validity in patients with PD [48].

### 2.6. Statistical Analysis

Due to the small sample size, continuous and ordinal variables were presented as medians with interquartile ranges, and non-parametric tests were used for statistical analysis. Categorical variables were presented as frequencies. First, patients with PD were categorized into two groups based on their age at the time of starting LSVT^®^ BIG: the older group (≥65 years) and the younger group (<65 years), following the definition set by the National Institute on Aging [49]. Next, before intervention, demographic characteristics were compared between the groups using the Wilcoxon rank-sum test for continuous and ordinal variables, and Fisher’s exact test for categorical variables. Finally, for the main analysis of clinical assessments, changes before and after intervention were compared between the older and younger groups using a two-way mixed-design analysis of variance (ANOVA) with an aligned rank transformation (ART) [50]. This analysis tested the main effects of evaluation timepoint (before vs. after intervention) and group (older vs. younger), as well as their interaction. If an interaction was identified, post hoc analyses were conducted using the Wilcoxon rank-sum test for between-group comparisons and the Wilcoxon signed-rank test for within-group comparisons. All statistical analyses were performed using R software (version 4.1.0; R Foundation for Statistical Computing, Vienna, Austria). Statistical significance was set at *p* < 0.05.

## 3. Results

### 3.1. Study Population

**Figure 1:**
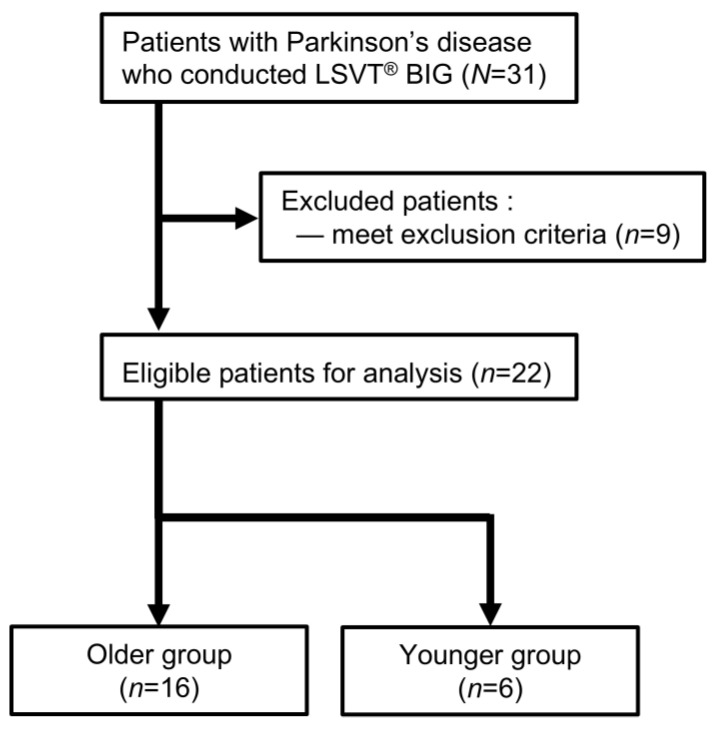
Flowchart of patient enrollment. Abbreviations: LSVT^®^, Lee Silverman Voice Treatment^®^.

A total of 31 inpatients and outpatients with PD who underwent LSVT^®^ BIG were screened from the Kawamura Hospital database, resulting in the selection of 22 eligible patients for analysis (Figure 1). The total number of eligible patients was within the range reported in similar previous studies [33,34,35,36]. Sixteen patients were classified as the older group, while the remaining 6 patients were classified as the younger group (Figure 1).

### 3.2. Demographic Characteristics Before Intervention

The demographic characteristics are shown in Table 1. The age of the older group was significantly higher than that of the younger group (*p* = 0.031). In contrast, no significant differences were observed between the older and younger age groups in terms of sex, H&Y stage, LEDD, time since PD diagnosis, number of inpatients versus outpatients, MMSE score, or total score of MDS-UPDRS Part I (*p* > 0.05).

### 3.3. Changes in Clinical Assessments Before and After the Intervention

**Figure 2:**
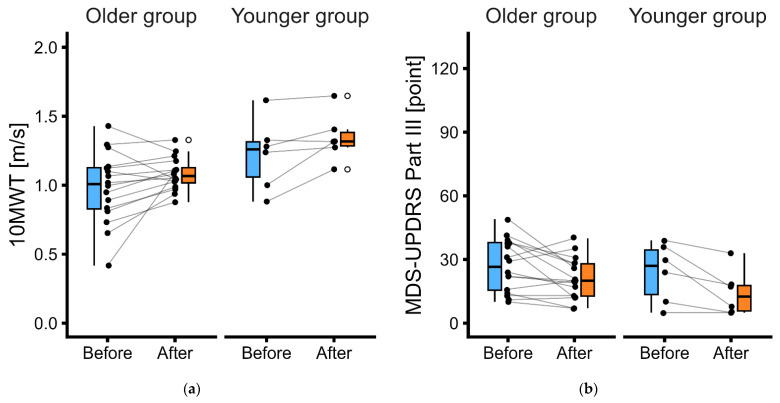

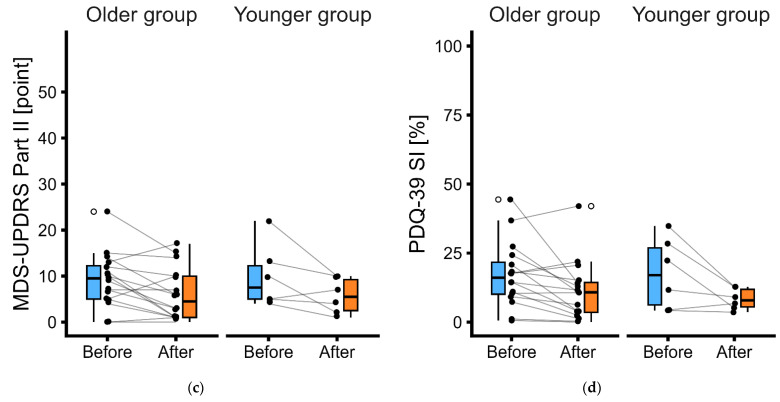
Changes in clinical assessments ((**a**) 10MWT, (**b**) MDS-UPDRS Part III, (**c**) MDS-UPDRS Part II, (**d**) PDQ-39 SI) before and after the intervention in the older and younger groups. The bold line within each box indicates the median. The lower end of the box represents the 25th percentile, while the top represents the 75th percentile. The lower whisker indicates the minimum value within the range of the 25th percentile minus 1.5 times the IQR, and the upper whisker indicates the maximum value within the range of the 75th percentile plus 1.5 times the IQR. White circles outside the whiskers represent outliers. Black circles represent individual patients, with lines connecting the before and after evaluations for the same individual. Panels display results for the older (**left**) and younger (**right**) groups. Abbreviations: 10MWT, 10-m walking test; MDS-UPDRS, Movement Disorder Society-sponsored revision of the Unified Parkinson’s Disease Rating Scale; PDQ-39 SI, Parkinson’s Disease Questionnaire-39 Summary Index; IQR, interquartile range.

Figure 2 illustrates the clinical assessment results before and after the 4-week LSVT^®^ BIG intervention. The results of the two-way mixed-design ANOVA with ART are presented in Table 2. The statistical analysis revealed a significant effect of the evaluation timepoint (before- vs. after-intervention), which was one of the main effects observed for all indicators (gait speed, motor symptoms, ADLs, and QoL). Regarding the other main effects, a significant effect of groups was observed in the 10 MWT. The interaction between the evaluation timepoint and group did not show a significant difference for any indicator.

## 4. Discussion

This study investigated the impact of age on the effectiveness of LSVT^®^ BIG on gait speed, motor symptoms, ADLs, and QoL in patients with PD. We used retrospective data collected from 2019 to 2026. Since all physical and occupational therapists were certified in LSVT^®^ BIG and delivered the intervention in accordance with the established protocol, the content and quality of the interventions were highly consistent. LSVT^®^ BIG, incorporating task-based interventions, improved gait speed, motor symptoms, ADLs, and QoL in patients with PD, without being affected by age differences between the elderly group (65 years and older) and the younger group (under 65 years). The minimal clinically important difference (MCID) of the gait speed, the total score of MDS-UPDRS Part III, and the PDQ-39 SI score were 0.02–0.10 m/s [51], −3.25 points [52], and −4.72% [53], respectively; these values were exceeded in both the older and younger groups. The MCID of the total score of MDS-UPDRS Part II was −3.05 points [54]; this threshold was exceeded in the older group but was not fully reached in the younger group.

Advanced age in patients with PD is associated with more severe motor symptoms, particularly balance and gait impairments [21,22,23]. LSVT^®^ BIG is designed to enhance self-perception of motor dysfunction and facilitate self-correction by increasing movement amplitude to counteract bradykinesia, hypokinesia, and akinesia [31,32,33]. To achieve this goal, repetitive task-based functional interventions were performed while adjusting task difficulty (e.g., use of handrails, sitting, or lying down) when patients were unable to perform tasks in the usual manner because of limited balance and/or gait ability. A systematic review and meta-analysis of randomized controlled trials reported that task-oriented exercise resulted in clinically meaningful improvements in gait speed in people aged 60 years and older, particularly those aged 75 years and older [55]. This approach works because task-oriented exercise facilitates motor learning by reinforcing correct movement patterns through repetitive functional tasks [55]. Two-way mixed-design ANOVA with ART revealed that gait speed was consistently slower in the older group than in the younger group both before and after the intervention. As an impact of aging, physical fitness and performance in terms of muscle volume, strength, flexibility, speed, and endurance decline [24,25]. Even under such differing conditions, LSVT^®^ BIG may nonetheless improve gait speed similarly across age groups.

With respect to ADL improvements, the LSVT^®^ BIG protocol may contribute to effectiveness in patients with PD across different age groups. Previous studies have reported that ADL difficulties vary with age and severity of motor symptoms [56,57]. Importantly, motor symptoms in patients with PD are associated with ADL independence, including feeding, bladder control, bathing, dressing, and mobility. Patients with PD and postural impairment experience significantly greater difficulty with feeding, toileting, bathing or showering, dressing or grooming, and mobility compared with those without postural impairment [56]. In addition, individuals aged 50–74 years experience difficulties with dressing and transferring, whereas those aged 75 years and older experience greater difficulties with bathing, dressing, and walking [57]. To address these age-specific challenges, the LSVT^®^ BIG protocol includes task-based ADL exercises tailored to individual needs and preferences, with adjusted difficulty levels to facilitate conscious use of large movements in daily life beyond the treatment setting [32]. Overall, co-designing meaningful ADL programs in partnership with patients, which enhance motivation and promote exercise adherence [58,59,60], has been shown to improve ADL performance.

Regarding QoL improvements, although PDQ-39 SI scores improved similarly in both age groups, the underlying factors contributing to these improvements may differ. Previous studies have reported that older patients with PD face challenges distinct from those of younger patients [61,62,63]. In particular, older patients experience greater negative impacts on mobility and self-care ability, contributing to QoL declines [61,62,63]. In this study, LSVT^®^ BIG may have improved gait ability, motor symptoms, and ADLs, thereby contributing to improved QoL in older patients with PD. In contrast, younger patients with PD have been reported to experience poorer emotional well-being than older patients [64,65]. Importantly, daily time spent being physically active has been associated with positive subjective states, such as happiness, motivation, and concentration [66]. Ehlen et al. (2018) reported that patients should be encouraged to increase activity levels through specialized programs that enhance self-efficacy and daily physical activity [66]. Building on this principle, LSVT^®^ BIG involves supervised one-hour sessions four times per week for 4 weeks, daily homework exercises, and patient-selected tasks guided by therapists. Notably, selecting meaningful tasks increases motivation and enhances a sense of achievement [58]. Furthermore, physical, instructional, and cognitive support; adherence reminders; and real-time feedback from interventionists have been shown to increase motivation and improve exercise adherence [59].

This study has some limitations. First, it included a small cohort from a single center and employed a retrospective and non-controlled design. Although the total number of eligible patients was within the range reported in similar previous studies [33,34,35,36], the number of patients in the younger group was relatively small. Second, the inclusion of inpatients in each group may have influenced results due to greater schedule control and potential monitoring or reinforcement of homework. Third, this study used patient-reported measures such as MDS-UPDRS Part II and PDQ-39 SI; these might have resulted in a few biases (e.g., recall bias or language bias) [67]. In addition, some assessment batteries used in this study were ordinal-scale data. Therefore, future prospective multicenter studies with larger sample sizes, using performance-based assessments to obtain ratio-scale data, are needed to confirm these findings and to further examine the effectiveness of task-based interventions in patients with PD across different age groups.

## 5. Conclusions

LSVT^®^ BIG may improve gait speed, motor symptoms, ADLs, and QoL in patients with PD, without being affected by age. This study holds significant clinical implications, suggesting that integrating such interventions into treatment plans for both inpatients and outpatients could benefit older and younger patients with PD. However, to confirm its effectiveness, studies with larger cohorts are warranted.

## Figures and Tables

**Table 1:** Participants’ demographic characteristics.

	Older Group (*n* = 16)	Younger Group (*n* = 6)	*p*-Value
Age (year)	74.0 [69.8, 77.0]	62.0 [57.8, 64.0]	***p* = 0.031** *^,a^
Sex [Male/Female]	[5/11]	[3/3]	*p* = 0.624 ^b^
H&Y stage [I/II/III/IV]	[0/5/5/6]	[1/2/2/1]	*p* = 0.462 ^b^
LEDD (mg/day)	625.0 [400.0, 872.3]	575.0 [362.5, 675.0]	*p* = 0.219 ^a^
Time since PD diagnosis (yrs)	4.5 [3.0, 7.0]	5.5 [2.5, 7.0]	*p* = 0.750 ^a^
Number of inpatients/outpatients	[2/14]	[1/5]	*p* = 1.000 ^b^
MMSE score	26.0 [24.0, 28.0]	30.0 [24.0, 30.0]	*p* = 1.000 ^a^
Total score of MDS-UPDRS Part I	6.0 [3.0, 9.3]	5.0 [3.5, 5.8]	*p* = 0.188 ^a^

Values are presented as median values [interquartile range]. Statistically significant results are indicated by bold values. * *p* < 0.05. ^a^ Wilcoxon rank-sum test. ^b^ Fisher’s exact test. Abbreviations: H&Y: Hoehn and Yahr; LEDD: levodopa equivalent daily dosage; MMSE: Mini-Mental State Examination; MDS-UPDRS: Movement Disorder Society-sponsored revision of the Unified Parkinson’s Disease Rating Scale; PD: Parkinson’s disease.

**Table 2:** Results before and after 4 weeks of LSVT^®^ BIG.

Assessment	Group	Before Intervention	After Intervention	Two-Way ANOVA		
				*p*-Value		
				Evaluation Timepoint	Group	Interaction
10MWT (m/s)	Older	1.0 [0.8, 1.1]	1.1 [1.0, 1.1]	***p* = 0.004** *	***p* = 0.016** *	*p* = 0.562
	Younger	1.3 [1.1, 1.3]	1.3 [1.3, 1.4]			
Total score of MDS-UPDRS Part III	Older	26.5 [15.5, 38.0]	20.0 [12.8, 28.0]	***p* = 0.002** *	*p* = 0.358	*p* = 0.515
	Younger	27.0 [13.5, 34.5]	12.5 [5.8, 17.8]			
Total score of MDS-UPDRS Part II	Older	9.5 [5.0, 12.3]	4.5 [1.0, 10.0]	***p* = 0.016** *	*p* = 0.860	*p* = 0.624
	Younger	7.5 [5.0, 12.3]	5.5 [2.5, 9.3]			
PDQ-39 SI (%)	Older	16.0 [10.1, 21.7]	10.8 [3.5, 14.3]	***p* = 0.003** *	*p* = 1.000	*p* = 0.781
	Younger	17.0 [6.2, 26.9]	7.9 [5.5, 11.8]			

Values are presented as median values [interquartile range]. Statistically significant results are indicated by bold values. * *p* < 0.05. Two-way mixed-design ANOVA, with ART. Abbreviations: ANOVA: analysis of variance; ART: Aligned Rank Transform; MDS-UPDRS: Movement Disorder Society-sponsored revision of the Unified Parkinson’s Disease Rating Scale; PDQ-39 SI: Parkinson’s Disease Questionnaire-39 Summary Index; 10MWT: 10-m walking test.

## Data Availability

The original contributions of this study are included in the article. Further inquiries can be directed to the corresponding author.

## Abbreviations

The following abbreviations are used in this manuscript:

PD	Parkinson’s disease
PIGD	Postural instability and gait disorders
ADL	Activities of daily living
QOL	Quality of life
LSVT^®^ BIG	Lee Silverman Voice Treatment^®^ BIG
H&Y	Hoehn and Yahr
MDS-UPDRS	Movement Disorders Society-Unified Parkinson’s Disease Rating Scale
LEDD	Levodopa equivalent daily dose
MMSE	Mini-Mental State Examination
10MWT	10-m walking test
PDQ-39 SI	Parkinson’s Disease Questionnaire-39 Summary Index
IQR	Interquartile range
ANOVA	Analysis of variance
ART	Aligned rank transformation
